# Opioid Use in Fibromyalgia Is Associated with Negative Health Related Measures in a Prospective Cohort Study

**DOI:** 10.1155/2013/898493

**Published:** 2013-03-18

**Authors:** Mary-Ann Fitzcharles, Neda Faregh, Peter A. Ste-Marie, Yoram Shir

**Affiliations:** ^1^Division of Rheumatology, McGill University, Montreal, QC, Canada H3G 1A4; ^2^Alan Edwards Pain Management Unit, Montreal General Hospital, McGill University Health Centre, 1650 Cedar Avenue, Montreal, QC, Canada H3G 1A4; ^3^Faculty of Law, University of Montreal, Montreal, QC, Canada H3C 3J7

## Abstract

As pain is the cardinal symptom of fibromyalgia (FM), strategies directed towards pain relief are an integral component of treatment. Opioid medications comprise a category of pharmacologic treatments which have impact on pain in various conditions with best evidence for acute pain relief. Although opioid therapy other than tramadol has never been formally tested for treatment of pain in FM, these agents are commonly used by patients. We have examined the effect of opioid treatments in patients diagnosed with FM and followed longitudinally in a multidisciplinary pain center over a period of 2 years. In this first study reporting on health related measures and opioid use in FM, opioid users had poorer symptoms and functional and occupational status compared to nonusers. Although opioid users may originally have had more severe symptoms at the onset of disease, we have no evidence that these agents improved status beyond standard care and may even have contributed to a less favourable outcome. Only a formal study of opioid use in FM will clarify this issue, but until then physicians must be vigilant regarding the multiple adverse consequences of opioid therapy.

## 1. Introduction

Chronic widespread pain is the pivotal symptom of fibromyalgia (FM). In the composite score that has been proposed for the new 2010 diagnostic criteria for FM, pain has been weighted to signify two thirds of the symptom component, with other symptoms including fatigue, sleep disturbance, cognitive changes, and somatic symptoms combined to represent the remaining one third of the symptom complex [1]. It is therefore logical that treatments directed towards pain relief will be an integral part of FM care. Traditional pharmacological treatments for managing pain are centered on simple analgesics, nonsteroidal anti-inflammatory drugs, and the opioid group of medications. Other than tramadol, opioids have never been formally studied as a therapeutic modality in FM and reports of efficacy are based solely on anecdotal and patient report [2]. Opioids are therefore not recommended by any current guidelines for the treatment of FM symptoms. 

Even in the absence of evidence for effect in FM, about 30% of Canadian and American FM patients reportedly using opioids [3, 4]. Opioids are also perceived by patients to offer the best symptom relief according to an internet survey of persons with self-reported FM [2]. There is however increasing concern regarding the negative effects associated with chronic opioid use, including an increased death rate especially in association with other agents such as benzodiazepines and alcohol [5]. We have recently reported the association of poorer health, psychosocial status, as well as substance abuse in patients carrying the label of FM and using opioids [3]. 

The aims of this study were to prospectively examine disease related measures in FM patients attending a multidisciplinary pain clinic, stratified according to opioid use at followup, and to evaluate the associations of opioid use including health related effects and psychosocial status. 

## 2. Materials and Methods

### 2.1. Design

We have examined opioid use and recorded outcome in a cohort of FM patients who are followed prospectively in a multidisciplinary pain clinic. This study, wherein drug use was openly observed, received ethics approval by the Ethics Committee of the Montreal General Hospital. The study cohort has previously been described [3]. 

### 2.2. Patients

All patients in this cohort were referred to the Alan Edwards Pain Management Unit from January 2005 with continued entry. Patients were entered into the cohort once the diagnosis of FM was confirmed by the study rheumatologist. All patients received information regarding good health lifestyle practices with recommendations for regular exercise activity and good eating habits with attention to weight control and were encouraged to develop a strong internal locus of control by being active participants in their healthcare. They were assessed and counselled by a psychologist experienced in the care of patients with chronic pain. Specific treatment strategies, including further psychological/behavioural interventions, rehabilitation program participation, and pharmacological interventions, were tailored according to individual patient symptoms and needs, taking into account previous treatment interventions. The frequency of follow-up visits for individual patients was dependent upon physician judgement.

### 2.3. Measurements

Baseline data included demographic, disease related, and psychosocial information. Demographic information included age, gender, education level, marital status, current employment status, and current disability payments. Information regarding symptom and functional status was recorded at entry and at a study followup during which all questionnaires were completed on a second occasion, which occurred at least 1 year after the baseline visit. The number of medications used was recorded and, if opioids were used, the morphine equivalent dose was calculated. Symptom and disease related information included measurements of pain, quality of life, function, and mood. 

#### 2.3.1. Measurements of Pain

Current pain was measured by a 10 cm visual analog scale (VAS). Pain quality was measured with the McGill Pain Questionnaire (MPQ) [6]. This is a validated questionnaire comprising 78 descriptor words arranged into 20 subgroups and measuring the sensory, affective, evaluative, and miscellaneous components of pain. Patients are asked to select the words which most accurately describe their pain. The total MPQ intensity score, with a maximum of 78, is calculated by summing the total number of words weighted by each word's rank order within its subcategory.

#### 2.3.2. Measurements of Quality of Life and Function

A patient global assessment (PGA) of disease was measured by a 10 cm VAS. Quality of life and function was measured by the Fibromyalgia Impact Questionnaire (FIQ) [7]. This is a condition specific, reliable, and validated measure for patients with FM. It consists of 19 subscales assessing physical function, number of days feeling bad, work missed, job ability, pain, fatigue, morning tiredness, stiffness, anxiety, and depression. The total score is out of 100, with higher score representing more severe functional impairment. Function was measured by the Health Assessment Questionnaire (HAQ), a generic questionnaire that measures outcome in patients with rheumatic diseases, validated for use in fibromyalgia [8]. 

#### 2.3.3. Psychological Variables

 Mood was assessed using the Arthritis Impact Measurement Scale (AIMS) for anxiety and depression [9]. Catastrophizing due to pain was measured with the Pain Catastrophizing Scale (PCS), a 13-item scale that addressed thoughts and feelings related to pain [10]. 

#### 2.3.4. Opioid Usage

Opioid use was recorded at baseline and at followup, with calculation of the morphine equivalent dose for opioids using the following morphine equivalent conversion factor: 10 mg morphine was equivalent to tramadol 100 mg, codeine 100 mg, hydromorphone 2 mg, oxycodone 5 mg, and meperidine 100 mg. The conversion for fentanyl patch was calculated as 25 mcg/hour being equivalent to morphine 75 mg per day. Methadone in a dose of <30 mg/day was calculated at 2.5 mg equivalent to morphine 10 mg and >30 mg/day as 1.5 mg equivalent to morphine 10 mg. 

Patients were categorized into 2 groups according to opioid usage at followup: Group 1 comprised all patients who were using opioids at the follow-up visit which included patients who entered the study on opioids and continued opioid treatments as well as those who initiated and continued opioids during the study period; Group 2 comprised all patients who were not using opioids at the follow-up visit which included those who at entry were not on opioids, those who discontinued opioids during the course of followup, and those who used opioids for a short time during the study period, but did not continue opioid treatment. 

### 2.4. Statistical Methods

The baseline data of the groups were tested to establish lack of methodological bias for pain, function, and psychological variables, by comparing between group mean differences for continuous variables and using chi-square tests for categorical variables. General linear model mixed repeated measures ANOVAs were conducted to assess whether there were significant differences in opioid status and pain, function, or psychological variables among patients at followup and whether there were significant interactions among these variables at two levels of the dependent variables (on opioids at followup and off opioids at followup). Multivariate Wilk's Lambda tests were used to assess the main effects of time. Between subject ANOVAs were used to test the main effects of opioid status followed by tests of opioid status by time interaction effects. Binary logistic regression tests were carried out to determine the statistical significance and degree of change in odds of group membership (on or off opioids) at followup for outcome variables. IBM SPSS Statistics version 19.0 was used to carry out the analyses.

## 3. Results

Of the 159 patients who entered the study, 131 (82%) had at least one followup visit at a mean ± SD of 26 ± 15 months. Their mean age was 50 ± 10 and 92% were females. The demographic and disease related variables of the 28 patients not seen in followup were not significantly different from the 131 patients in this study cohort. Demographic and disease related information for all 131 patients, as well as for the subdivision into Group 1 (opioid users) and Group 2 (nonopioid users), is shown in Table 1. At baseline there were no significant differences for any demographic variables between Groups 1 and 2. Generally, scores for disease related variables were higher for Group 1 versus Group 2 at baseline (Table 1). Similarly, at followup, the mean values for multiple measures of symptoms were higher for opioid users, demonstrating that the latter are more symptomatic (Table 2). 

At entry, 34 patients were using an opioid. Fifteen were using a weak opioid, either codeine or tramadol, and 19 were using strong opioids with an average morphine equivalent dose of 50 mg. Twelve patients in Group 2 were given a trial of opioids, followed by discontinuation. At the recorded follow-up visit, 43 (33%; Group 1) patients were on opioids, 20 of whom had continued treatment from entry and 23 of whom had an opioid treatment initiated during the follow-up period, and 88 (67%; Group 2) patients were not using opioids at followup.

Sixteen of the 43 opioid users were on either tramadol or codeine, and 27 were on a strong opioid. Four patients were using 2 different opioids, each were on a long acting and a short acting agent. The mean morphine equivalent dose of opioid at followup was 48 mg a day. When opioid users were categorized according to use of tramadol or methadone in one group (23 patients) and other opioids in a second group (20 patients), there was a trend for those using tramadol or methadone to score better for all outcome measures even though the morphine equivalent dose of 48 mg versus 47 mg was similar (data not shown). 

There was a significant main effect of change over time for pain, function, and mood variables within the total cohort (Table 3). Changing scores from baseline to followup were significantly different for pain as measured by the pain VAS and MPQ, for function as measured by the FIQ, and for mood as measured by the AIMS depression and anxiety and the PCS. There was a significant main effect of opioid use for pain (pain VAS, MPQ) and function (PGA, FIQ, and HAQ) variables, but not for mood. There were no signification interaction effects for change over time by opioid status for measures of pain, mood, and function with the exception of the HAQ (*F* = 6.72, *P* = 0.011, Eta = 0.22).

The results of the binary logistic regressions showed that, compared to those not on opioids at followup, patients who took opioids were significantly more likely to be on disability (OR = 0.36, 95% C.I. = 0.151–0.855) and more than twice as likely to be unemployed (OR = 2.29, 95% C.I. = 1.001–5.215).

## 4. Discussion

We have observed that one third of FM patients followed longitudinally in a multidisciplinary pain clinic were maintained on opioid drug therapy. Over time, there was an improvement recorded for the total cohort for measures of pain, function, and mood, irrespective of opioid status. However, opioid users scored consistently higher for all measures of symptom severity with significance noted for higher pain scores and more functional impairment. The only significant interaction between time and opioid status was noted for the single measurement of physical function, the HAQ, with a small effect size. These results suggest that although function, pain, and psychological variables improved during care in a multidisciplinary pain clinic, these measures were independent of opioid use. Importantly, opioid use was not associated with disease status improvements beyond that seen for standard care in a multidisciplinary setting. Additionally, work status of opioid users was less favourable with more unemployment and disability payments noted for the opioid group. The findings of this open observational study raise questions and concerns regarding the rational use of opioid treatments in FM patients. 

There is currently no optimal treatment for FM, although the current concept is to direct treatments towards specific symptoms, with the ideal pharmacotherapy addressing more than one symptom [11]. Pain is a recognized important component of FM, impacting on global wellbeing and function. Nevertheless, the role of the endogenous opioid system in pain expression in FM is unclear, with studies reporting conflicting results of down- and upregulation of opioid receptors, elevated levels of enkephalins in the cerebrospinal fluid and variable response to the opioid antagonist naltrexone [12, 13]. Pain response is currently used as a relevant outcome measurement for assessment of treatments in clinical trials in FM [14]. Efficacy of any treatment strategy in FM should however reflect both improvements in the target symptom(s) as well as function. Therefore, a treatment with a major effect on pain relief should ideally be associated with improvement across a wider range of outcome measures and especially with functional status. 

Use of opioids has been entrenched in the management of chronic pain conditions. Our findings of 30% prevalence in use of opioids in FM patients are in line with reports of opioid use in patients with chronic nonmalignant painful conditions [15]. Indeed, the use of any analgesic other than nonsteroidal anti-inflammatory drugs was reported by over half of FM patients in a survey in the United States [4]. It is also notable that FM patients identify opioids as the category of drugs offering best symptom relief [2]. An Internet survey of more than 2000 FM patients showed that hydrocodone and oxycodone were amongst the medications perceived as most effective [2]. In this internet study, FM patients also identified benzodiazepines as effective therapy, another group of drugs associated with potential for harm.

While the place of opioid therapy, particularly the use of strong opioids, in the management of FM pain remains controversial, the known analgesic properties of these agents should encourage further study in FM. Opioid medications offer the best available short-term pharmacologic analgesia for almost any pain, although opioids are frequently discontinued in treatment trials of the management of chronic pain [16]. In our study the morphine equivalent dose of opioids at entry and at followup was in the order of 50 mg per day. This is considered to be a moderate dose and is clearly in the range that could be associated with side effects. Only a third of those using opioids were using weak opioids, either codeine or tramadol. To date, only tramadol has been formally studied in FM, with a positive effect on pain and quality of life [17, 18]. It is notable that patients treated with either tramadol or methadone showed a nonsignificant trend for a better outcome than those treated with other opioids, even with an equivalent morphine dose for both groups, suggesting that the different mechanisms of action for these two agents may have clinical implications requiring further study. Opioid use is however not supported by recommendations from any guidelines for the management of FM [19–21]. 

In that patients on opioid treatments fared worse than those not on opioids, a number of factors require consideration. It is possible that those patients maintained on opioids had an overall more severe disease process or had more comorbidities contributing to continued opioid use as has been previously reported [22]. Although demographic variables did not differ when baseline information was compared between patients stratified according to opioid status at followup, it is notable that the opioid group did have more severe pain, functional impairment, and mood disorder at entry compared to the nonopioid group. This might suggest that those using opioids were more symptomatic from outset. In view of a mean duration of disease that was greater than a decade for this cohort, we are unable to make any statement regarding severity of disease at onset. Although the two groups did not differ demographically, those using opioids generally scored higher for all disease related variables, indicating more suffering. Rather than showing a better clinical status associated with opioid use, we have observed that opioid users, even when followed longitudinally by a multidisciplinary team, were more symptomatic and functionally impaired, raising the question whether opioids per se may have contributed to these findings due to the effects of increasing pain associated with central sensitization [23]. This suggestion is plausible as there are considerable similarities between symptoms of FM and side effects related to opioid use, such as fatigue, poorer physical and mental wellbeing, and even increasing pain. These negative effects may also be compounded by medications with psychoactive effects such as tranquillizers, antidepressant medications and anticonvulsants. Without improvement in function, or at the very least maintenance of function, opioid therapy in particular as well as other pharmacologic therapy should be critically evaluated and continued treatment justified. 

Because FM symptoms tend to persist over time and only rarely are completely resolved, pharmacologic therapies will necessarily be used over prolonged periods requiring rigorous evaluation of risk benefit ratio. There has been increased concern regarding the immediate as well as the long term side effects of opioid treatments in an individual patient and also societal concerns about the misuse and abuse of prescription opioids. The long term effects of chronic opioid use in nonmalignant pain are not yet fully clarified, but effects on mood, cognitive function, hormonal effects, and increased pain due to hyperalgesia need to be constantly reevaluated [24]. We have previously shown in a cross sectional study that opioid use was associated with negative psychosocial effects including unstable psychiatric disorder, history of substance abuse, unemployment, and disability payments in patients carrying the diagnosis of FM, but in whom only 66% were confirmed as having FM [3]. Our original observations raised the concern that some persons could even be misusing the diagnosis of FM in order to procure prescription medication such as opioids. 

The progressive increase in opioid prescriptions has seen a parallel increase in their use as drugs of abuse [5, 25–27]. There are reports of increased deaths associated with overdose of opioids, usually in younger individuals, often receiving prescription opioids and usually combined with other agents such as alcohol or benzodiazepines. Guidelines for safe and effective use of opioids for chronic pain have been published by the American Pain Society and also in Canada [24, 28]. The tone of both guidelines is cautious, urging physicians to practice responsible prescribing behaviours, pay attention to physical and psychosocial aspects of patient care, and constantly reevaluate the efficacy and side effect profile of prescribed opioids. 

### 4.1. Limitations

A number of limitations of this study are acknowledged. Firstly, most patients recruited to this study already had symptom duration for over 10 years. We are therefore unable to make any comment regarding the severity of symptoms either at onset of disease or in the absence of treatments. Secondly, as this is a real life study with patient tailored treatments, there was no predetermined protocol to dictate treatment choices; treatments were selected according to individual physician judgement, depending on the predominant symptoms in an individual patient. Thirdly, the duration of opioid treatments for those entering the study on opioids is unknown and, although opioid status at endpoint was recorded, we did not have sufficient information to report on overall duration of opioid use for individual patients. Finally, although wisdom suggests that opioid treatments may be less favourable for patients with FM, patient choices regarding treatments were respected and may have led to continued opioid treatments for some patients which may have been contrary to the recommendation of the multidisciplinary treating team. 

## 5. Conclusions

In this first study reporting on health related measures and opioid use in FM patients, followed longitudinally over a two year period, opioid treated patients were more symptomatic and were more likely to be unemployed and to be receiving disability benefits. While opioids remain a treatment choice for management of pain, we are concerned that our patients using opioids failed to show any advantage in overall health status. Although opioids may have been initiated due to more severe symptoms, we have no evidence that the addition of these agents to the standard care received in a multidisciplinary pain clinic improved disease status or function. Only a formal clinical trial of opioid use in FM will clarify this issue, but until then, we advise physicians to be vigilant regarding the need for continued treatment.

## Figures and Tables

**Table 1:** Demographic and disease related information at baseline for 131 patients stratified according to opioid use at followup.

	All	Group 1	Group 2
		Opioid users	Nonopioid users
	*n* = 131	*n* = 43	*n* = 88
Gender			
Female	120	40	80
Male	11	3	8
Age, mean ± SD	50 ± 10	50 ± 10	49 ± 10
Dur. pain, mean ± SD	11 ± 10	11 ± 10	11 ± 10
Education			
<High school, *n* (%)	15 (11)	5 (12)	10 (11)
High school, *n* (%)	43 (33)	14 (33)	29 (33)
College, *n* (%)	38 (29)	16 (37)	22 (25)
University, *n* (%)	35 (27)	8 (19)	27 (31)
Marital status			
Single, *n* (%)	26 (20)	5 (12)	21 (24)
Married, *n* (%)	83 (63)	32 (74)	51 (58)
Divorced, *n* (%)	15 (11)	4 (9)	11 (13)
Widowed, *n* (%)	5 (4)	1 (2)	4 (5)
Employed, *n* (%)	39 (30)	11 (26)	28 (32)
Disability, *n* (%)	42 (32)	16 (37)	26 (30)
Medications, *n* ± SD	2.3 ± 1.3	2.6 ± 1.3	2.2 ± 1.3
Pain			
Pain VAS, mean ± SD	6.4 ± 2.3	6.9 ± 2.2	6.2 ± 2.4
MPQ, mean ± SD	42 ± 15	46 ± 15*	40 ± 15
Function			
Patient global VAS, mean ± SD	6.4 ± 2.4	7.1 ± 2.3*	6.1 ± 2.4
FIQ, mean ± SD	66 ± 18	72 ± 15**	63 ± 18
HAQ, mean ± SD	1.14 ± 0.66	1.23 ± 0.67	1.09 ± 0.65
Mood			
AIMS anx, mean ± SD	6.1 ± 1.9	6.4 ± 1.7	6.0 ± 2.0
AIMS dep, mean ± SD	5.0 ± 1.6	5.2 ± 1.4	4.9 ± 1.7
PCS, mean ± SD	29 ± 12	30 ± 13	28 ± 12

Group 1 baseline versus Group 2 baseline: *P ≤ 0.05, **P ≤ 0.01.

SD: standard deviation, VAS: Visual Analog Scale, MPQ: McGill Pain Questionnaire, FIQ: Fibromyalgia Impact Questionnaire, HAQ: Health Assessment Questionnaire, AIMS: Arthritis Impact Measurement Scale, and PCS: Pain Catastrophizing Scale.

**Table 2:** Disease related information at baseline and followup for 131 patients stratified according to opioid use at followup.

	Group 1	Group 1	Group 2	Group 2
	Baseline *n* = 43	Followup *n* = 43	Baseline *n* = 88	Followup *n* = 88
	mean ± SD	mean ± SD	mean ± SD	mean ± SD
Pain				
Pain VAS	6.9 ± 2.2	6.5 ± 2.5*	6.1 ± 2.4	5.4 ± 2.9
MPQ	46 ± 15	43 ± 17**	40 ± 15	32 ± 17
Function				
PGA	7.1 ± 2.3	6.7 ± 2.7*	6.1 ± 2.4	5.5 ± 2.6
FIQ	72 ± 15	66 ± 20**	63 ± 18	54 ± 22
HAQ	1.23 ± 0.67	1.27 ± 0.71**	1.09 ± 0.65	0.87 ± 0.68
Mood				
AIMS anx	6.4 ± 1.7	5.6 ± 2.1	6.0 ± 2.0	5.4 ± 2.0
AIMS dep	5.2 ± 1.4	3.9 ± 2.1	4.9 ± 1.7	3.3 ± 1.8
PCS	30 ± 13	25 ± 14	28 ± 12	22 ± 14

Group 1 FU versus Group 2 FU: *P ≤ 0.05, **P ≤ 0.01.

SD: standard deviation, VAS: Visual Analog Scale, MPQ: McGill Pain Questionnaire, FIQ: Fibromyalgia Impact Questionnaire, HAQ: Health Assessment Questionnaire, AIMS: Arthritis Impact Measurement Scale, PCS: Pain Catastrophizing Scale.

**Table 3:** Results of Multivariate and Univariate tests of General Linear Model Repeated Measures.

Variable	Multivariate test—Wilk's Lambda—*F*			Between-Subject Effects—Main effect of Opioid status—*F*			Interaction Opioid status ∗ time		
Function	*F*	sig	Eta squared	*F*	sig	Eta squared	*F*	sig	Eta squared
FIQ	13.15	0.001	0.95	11.08	0.001	0.081	0.493	0.484	0.004
HAQ	3.651	0.58	0.028	5.246	0.024	0.039	6.716	0.011	0.049
Depression	50.169	0.001	0.280	2.554	0.112	0.019	0.538	0.464	0.004
Anxiety	13.167	0.001	0.093	0.800	0.373	0.006	0.181	0.671	0.001
VAS	4.420	0.037	0.033	5.434	0.021	0.041	0.632	0.428	0.005
PGA	2.264	0.135	0.017	8.231	0.005	0.060	0.377	0.540	0.003
McGill Total	13.102	0.001	0.093	9.905	0.002	0.072	2.046	0.155	0.016
Catastrophizing	24.048	0.001	0.159	1.015	0.316	0.008	0.295	0.588	0.002
